# Locked and loaded: targeting extracellular vesicles to preserve sight

**DOI:** 10.1038/s42003-023-05672-7

**Published:** 2023-12-18

**Authors:** Yvette Wooff

**Affiliations:** 1 John Curtin School of Medical Research, Eccles Institute of Neuroscience, Australian National University, Acton, ACT 2601 Australia; 2 School of Medicine and Psychology, Australian National University, Acton, ACT 2601 Australia

## Abstract

Retinal degenerative diseases are often multifaceted and difficult to treat, instead requiring more targeted or personalized therapeutic solutions. Recent work by Liu et al. reveals one such pipeline to engineer extracellular vesicles that can selectively reduce the spread of retinal inflammation and prevent the progression of vision loss in rodent models of retinal degeneration. This approach is representative of a new wave of precision medicines with the potential to treat these otherwise incurable diseases.

**Figure:**
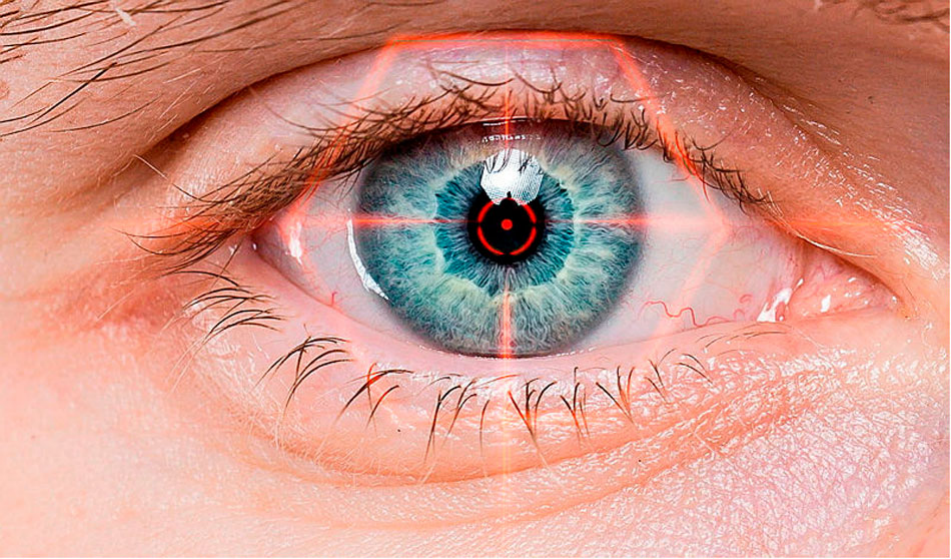
Oz, stock.adobe.com

Retinal degenerations are a heterogenous group of diseases which result in irreversible blindness and affect over 300 million people worldwide^1^, with no available treatments or cures. Since retinal degenerative diseases are often marked by high levels of oxidative stress and inflammation, dampening inflammatory signalling is a prominent therapeutic strategy. However, efforts to develop relevant drugs that combat widespread inflammation are complicated by the multifaceted nature of these diseases and the immune-privileged environment in which the retina resides^2^. The use of drugs that cannot easily transverse the blood-retina barrier, or synthetic drug carriers which may induce secondary inflammatory reactions, are therefore not suitable for treating retinal degenerative diseases^3^.

The use of extracellular vesicle (EV)-based therapeutics for the retina has gained serious momentum in recent years, owing to their biocompatible, nanosized, proliferative, and neuroprotective properties^4–7^. However, therapeutic loading and selective targeting of EVs to the site of damage are two significant hurdles preventing their use for treating retinal degenerations. Recent work by Liu et al.^8^, describes a method to target EVs derived from human umbilical mesenchymal stem cells to inflammatory microglial cells in the degenerating retina, by adding a modified cyclic RGD peptide on the EV surface. Further, these authors demonstrated that by additionally loading these targeted EVs with an anti-inflammatory drug (anakinra)^9,10^, they were not only able to selectively target retinal microglia, but were able to demonstrate improved therapeutic efficacy in protecting against retinal degeneration compared to both naïve EV and direct anakinra delivery. These results were seen in both MNU-induced degeneration and *rd1-*derived retinitis pigmentosa models of retinal degeneration. Importantly, the authors showed that the use of these targeted and loaded EV was able to dampen key inflammatory pathways known to be upregulated across retinal degenerative diseases, including Nf-κb, caspase-mediated apoptosis, and pro-inflammatory interleukin signalling^2^.

Overall, the strength of this study lies in its three-pronged approach of combining a well-established mesenchymal stem cell EV population^11,12^, loaded with an anti-inflammatory drug target, and selectively targeted to microglia - the inflammatory cells of the retina. While this approach was beneficial across in vivo mouse models of retinal degeneration, it is limited by the reliance on synthetic liposomes to create surface-modified targeted EV populations that might induce some level of inflammation in the retina^3,12^. Investigations into the safety of both the naked and loaded EV, would strengthen this manuscript as well as the translatable potential of this therapeutic strategy. Regardless, designer EVs for ophthalmic treatment still represent a much-needed step in the right direction to treat retinal degeneration and preserve the gift of sight for millions of patients.
